# Antiviral Activity and Increased Host Defense against Influenza Infection Elicited by the Human Cathelicidin LL-37

**DOI:** 10.1371/journal.pone.0025333

**Published:** 2011-10-21

**Authors:** Peter G. Barlow, Pavel Svoboda, Annie Mackellar, Anthony A. Nash, Ian A. York, Jan Pohl, Donald J. Davidson, Ruben O. Donis

**Affiliations:** 1 Influenza Division, Centers for Disease Control and Prevention, Atlanta, Georgia, United States of America; 2 Biotechnology Core Facility Branch, Division of Scientific Resources, Centers for Disease Control and Prevention, Atlanta, Georgia, United States of America; 3 MRC Centre for Inflammation Research, Queens Medical Research Institute, The University of Edinburgh, Edinburgh, United Kingdom; 4 The Roslin Institute and Centre for Infectious Diseases, University of Edinburgh, Edinburgh, United Kingdom; Oklahoma Medical Research Foundation, United States of America

## Abstract

The extensive world-wide morbidity and mortality caused by influenza A viruses highlights the need for new insights into the host immune response and novel treatment approaches. Cationic Host Defense Peptides (CHDP, also known as antimicrobial peptides), which include cathelicidins and defensins, are key components of the innate immune system that are upregulated during infection and inflammation. Cathelicidins have immunomodulatory and anti-viral effects, but their impact on influenza virus infection has not been previously assessed. We therefore evaluated the effect of cathelicidin peptides on disease caused by influenza A virus in mice. The human cathelicidin, LL-37, and the murine cathelicidin, mCRAMP, demonstrated significant anti-viral activity *in vivo*, reducing disease severity and viral replication in infected mice to a similar extent as the well-characterized influenza virus-specific antiviral drug zanamivir. *In vitro* and *in vivo* experiments suggested that the peptides may act directly on the influenza virion rather than via receptor-based mechanisms. Influenza virus-infected mice treated with LL-37 had lower concentrations of pro-inflammatory cytokines in the lung than did infected animals that had not been treated with cathelicidin peptides. These data suggest that treatment of influenza-infected individuals with cathelicidin-derived therapeutics, or modulation of endogenous cathelicidin production may provide significant protection against disease.

## Introduction

Infection with influenza viruses is a significant cause of morbidity and mortality. Vaccination can help protect against prevalent subtypes of influenza, but new subtypes represent a global pandemic threat, and emerging resistance to neuraminidase (NA) inhibitors (the current first-line therapy) is of serious concern. Thus, in addition to effective vaccination strategies, the development of novel, alternative therapeutics is of great importance.

Innate immune mechanisms are critical to the host response to respiratory infection with influenza virus [1]. As well as cytokines and chemokines, both cellular responses (such as neutrophils, macrophages, and NK cells) and other soluble factors found within airway surface liquid (such as defensins and collectins) have been shown to assist in the containment and clearance of an initial influenza infection [2], [3]. The effectiveness of these innate responses to virus at the primary site of infection is likely to be critical to the pathological outcomes of the disease, and targeting these responses may lead to novel therapeutic agents.

Important components of early innate immunity are cationic host-defense peptides (CHDP; also known as antimicrobial peptides). Although initially described primarily as antibacterial agents, we and others have characterised them as modulators of inflammation and immunity [4], [5], and recent studies have also demonstrated direct antiviral potential [6], [7], [8], [9], [10]. The two major families of CHDP in mammals are cathelicidins and defensins. Whereas the multitude of defensins and the very mild phenotypes observed in specific defensin-null mice [11], [12] suggests considerable functional redundancy, humans and mice express a single cathelicidin. Increased susceptibility to infection is observed in individuals with morbus Kostmann (in which neutrophils are cathelicidin-deficient) [13], and mice deficient in the murine cathelicidin mCRAMP/Camp have significantly increased susceptibility to bacterial infection in multiple systems, including the lung [14], [15], [16], [17], [18]. Although the precise mechanisms involved, and the relative roles of microbicidal activity and immunomodulatory functions remain unknown, these studies clearly demonstrate the importance of this CHDP in host defence against bacterial infection.

The sole human cathelicidin hCAP-18 (CAMP) is stored in neutrophil-specific granules and is inducible in epithelial cells and macrophages [19]. LL-37 is the predominant active CHDP, generated from hCAP-18 by proteinase-3 [20]. LL-37 can be detected in airway surface liquid, plasma, sweat and other body fluids, and is upregulated in response to infection and inflammation. Although the primary source of LL-37 is pre-stored hCAP-18 in neutrophil granules, inducible production in epithelial cells may be important in the earliest innate response to pathogens. Interestingly, the upregulation of LL-37 expression in epithelial cells and macrophages has been shown to be vitamin-D dependent [21], [22], [23], including in response to respiratory syncytial virus [21]. Vitamin D-dependent upregulation of LL-37 may therefore help explain the protective effect of vitamin D against viral infection [24], [25], raising the possibility of an anti-viral role for LL-37 in the lung.

Evidence for cathelicidin-mediated anti-viral functions have been provided by recent demonstrations of inhibition of HIV-1 replication [10], reduced vaccinia plaque formation *in vitro,* and increased susceptibility of cathelicidin-deficient mice to eczema vaccinatum following vaccinia virus inoculation [26]. Based on the antiviral activity of LL-37 and its importance in the response to lung infections, as well as the need for new antiviral drugs against influenza, this study assessed the potential for cathelicidins to exert antiviral effects against influenza virus in *in vitro* and *in vivo* models of infection.

## Results

### LL-37 Protects Mice Against Influenza Virus Disease

**Figure 1:**
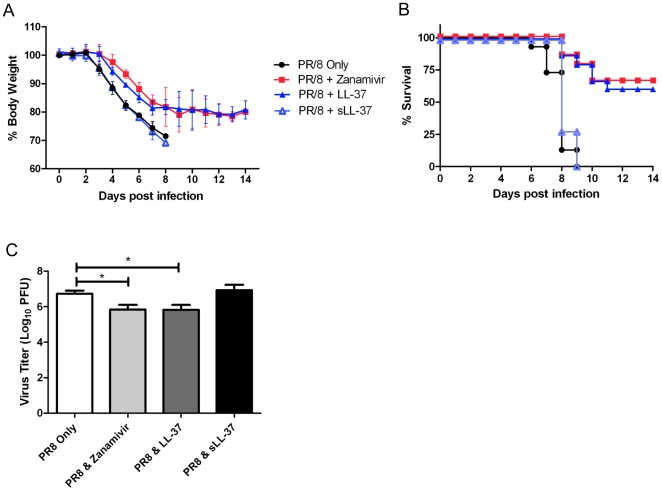
LL-37 Protects Mice Against Influenza Virus Disease. (A,B) Groups of 5 mice were inoculated with 10 MLD_50_ of A/Puerto Rico/8/1934 influenza virus by the intranasal route on day 0. Mice were nebulized with 200 µl of saline (control), zanamivir (500 µg/ml), LL-37 peptide (500 µg/ml) or scrambled LL-37 control peptide (500 µg/ml) once daily from day -1 to day 7. Mouse body weight (A) and survival (B) was monitored daily up to 14 days post infection. Data represent mean values ± SEM, for three independent experiments. Statistical analysis was performed using Kaplan Meier with a Mantel-Cox (log rank) test. Survival curves obtained with Zanamivir and LL-37 treatments were significantly different (P≤0.001) compared to saline control treatment. There was no difference between saline treated and sLL-37 treated groups. (C) Groups of three mice (Female, 6–8 week old Balb/c) were inoculated with 10 MLD_50_ of A/PR/8/34 virus intranasally on day 0. Mice were nebulized with 200 µl of saline (control) zanamivir (500 µg/ml), LL-37 peptide (500 µg/ml) or scrambled LL-37 control peptide (500 µg/ml) once daily from day -1 to day 2. Mice were euthanized on day 3 and viral titer in the lungs was assessed by plaque assay. Figure is representative of three independent experiments. Figure shows mean values ± SEM. Statistical analysis was performed using a Student t-test to compare virus infected animals with virus/peptide and virus/zanamivir treated animals (*P≤0.05).

To determine if LL-37 displayed antiviral activity in a murine model of lethal influenza virus infection, mice (6–8 week old, female Balb/C) were treated with nebulized LL-37 for 1 day prior to and for 7 days post infection with 10×50% Mouse Lethal Doses (MLD_50_) A/Puerto Rico/8/1934 (H1N1) virus (PR/8). Nebulized zanamivir and a scrambled LL-37 peptide were used as positive and negative controls respectively. Mouse body weight and survival were monitored for 14 days following infection. Mice infected with PR/8 and treated with nebulized saline or control scrambled peptide all showed severe disease with severe weight loss by day 8 post infection. In contrast, mice that received treatment with either LL-37 or the antiviral drug, zanamivir, showed significantly decreased body weight loss (P≤0.05 and P≤0.01 respectively) at 7 dpi (maximum weight loss observed was ∼20%). Weight loss in LL-37 and zanamivir treated mice ceased around day 9 (Figure 1A).

Mice infected with PR/8 and nebulized with either saline or scrambled peptide either died or were required to be euthanized due to severe disease 8–9 days after infection, whereas mice that had received either nebulized LL-37 or nebulized zanamivir showed a significant increase in survival (P≤0.001; Figure 1B). Survival of mice receiving LL-37 was comparable to that of mice receiving zanamivir (approximately 60% of mice survived in both treatments) clearly demonstrating that therapeutic use of LL-37 was able to reduce the severity of the influenza virus infection to a similar extent as the well characterized antiviral drug zanamivir, a current first-line human therapeutic.

In order to assess whether changes in disease severity could be attributed to a reduced viral load in the lungs, groups of mice were euthanized at 3 days post infection and lung virus titers were measured by plaque assay. Compared to control-treated mice, mice that had been treated with LL-37 showed significant reduction of approximately 70-80% in lung virus titer at 3 days post infection (p≤0.05), a similar level of reduction as seen after zanamivir treatment (Figure 1C). Treatment with control scrambled peptide had no effect on lung virus titers demonstrating that the protective response was not a non-specific effect of treatment with a charged peptide. These data demonstrate the antiviral potential of the therapeutic use of LL-37.

### Cathelicidins Show Species-Specific Antiviral Effects

**Figure 2:**
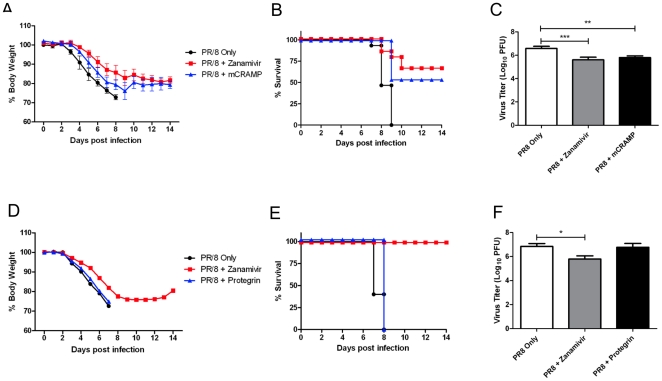
Cathelicidins Show Species-Specific Antiviral Effects. (A,B,D,E) Groups of 5 mice were infected with 10 MLD_50_ of A/PR/8/34 influenza virus via intranasal administration on day 0. Mice were nebulized with 200 µl of saline (control), zanamivir (500 µg/ml), the murine cathelicidin mCRAMP (500 µg/ml) (A and B) or the porcine cathelicidin Protegrin-1 (500 µg/ml) (D and E) once daily from day -1 to day 7. Mouse body weight (A, D) and survival (B, E) was monitored daily up to 14 days post infection. Data represent mean values ± SEM, for three independent experiments (A and B) or one experiment (D and E). Statistical analysis was performed using Kaplan Meier with a Mantel-Cox (log rank) test. Survival curves obtained with Zanamivir and mCRAMP treatments were significantly different (P≤0.001) compared to saline control treatment. There was no difference between saline treated and Protegrin treated groups. (C, F) Groups of three mice were infected with 10 MLD_50_ of A/PR/8/34 virus via intranasal administration on day 0. Mice were nebulized with 200 µl of saline (control), zanamivir (500 µg/ml), the murine cathelicidin mCRAMP (500 µg/ml) or the porcine cathelicidin Protegrin-1 (500 µg/ml) once daily from day -1 to day 2. Mice were euthanized on day 3 and viral titer in the lungs was assessed by plaque assay. Figure shows mean values ± SEM. Statistical analysis was performed using an unpaired t-test to compare virus infected animals with virus/peptide and virus/zanamivir treated animals (*P≤0.05, **P≤0.01, ***P≤0.001).

LL-37 is a human cathelicidin, although it has previously been shown to have antimicrobial effects in mouse models of infection [27]. To determine if other peptides of the cathelicidin family also conferred protection against influenza virus *in vivo*, mice were treated with the murine mCRAMP peptide or the porcine Protegrin-1 peptide for 1 day prior to and for 7 days post infection with 10 MLD_50_ A/PR/8/34 (H1N1) virus. Nebulized zanamivir was used as a positive control. As with LL-37, mice infected with PR/8 and nebulized with mCRAMP lost less weight when compared to control mice that had received only nebulized saline (Figure 2A). Treatment with mCRAMP also dramatically resulted in significantly enhanced survival (Figure 2B, p<0.001) and reduced lung virus titers by approximately 70–80% (Figure 2C). Animals treated with mCRAMP showed similar weight loss, survival, and lung virus titers as those treated with zanamivir (Figure 2A–C). In contrast, mice that had received treatments with nebulized Protegrin-1 peptide lost weight to the same extent as the control-treated animals (Figure 2D), and did not show any statistically significant increase in survival (Figure 2E), or reduction in lung virus titers (Figure 2F).

These data further demonstrate the specificity of the anti-viral effect between cathelicidins from divergent species. They also indicate that murine and human cathelicidins may play a significant role in innate host defense against influenza infection and demonstrate the capacity to enhance this defense with increased levels of native cathelicidins.

### Cathelicidins Mediate Changes in Lung Cytokine Concentrations Following Influenza Virus Infection

**Figure 3:**
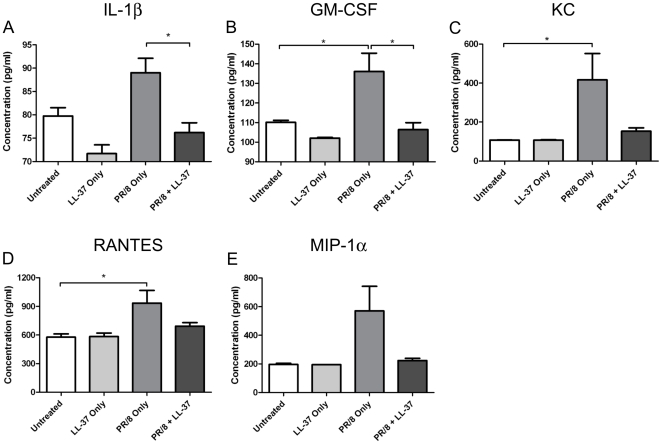
Cathelicidins Mediate Changes in Lung Cytokine Concentrations Following Influenza Virus Infection. Groups of 5 mice were infected with 10 MLD_50_ of A/PR/8/34 influenza virus via intranasal administration on day 0. Mice were nebulized with 200 µl of saline (control), or LL-37 peptide (500 µg/ml) once daily from day -1 to day 2. Mice were euthanized on day 3 and concentration of the indicated cytokines in the bronchoalveolar lavage (BAL) fluid were measured by BioPlex assay. Figures show mean values ± SEM. Statistical analysis was performed using an two-way analysis of variance (ANOVA) (*P≤0.05).

Cathelicidins have been shown to elicit a number of protective mechanisms in host defense against infection. To determine whether LL-37 might be protecting against influenza infection by modulating the inflammatory response in the lung, a number of cytokines in the bronchoalveolar lavage (BAL) fluid of mice were assessed using a BioPlex assay. Groups of 5 mice were treated with nebulized LL-37 or with saline for 1 day prior to and 2 days after infection with A/PR/8/34 (H1N1). Other groups were not infected and received either LL-37 only or saline only. After PR/8 infection, a number of inflammatory cytokines were found to be increased in the BAL fluid of control infected mice. Significant (p≤0.05) increases of IL-1β, GM-CSF, KC and RANTES were observed in the BAL fluid three days after infection. Treatment with LL-37 did not significantly alter cytokine concentrations in the absence of influenza virus infection. However, in infected mice treated with LL-37 the BAL fluid concentrations of IL-1β, GM-CSF, KC and RANTES were found to be unchanged compared with uninfected controls, and IL-1β and GM-CSF were significantly lower (p≤0.05) than in PR/8 infected control animals. Mice infected with PR/8 also showed a non-significant increase in the BAL fluid concentration of MIP-1α compared to naïve untreated animals (Figure 3E) and this increase was ameliorated when mice were nebulized with LL-37.

These data demonstrate the capacity of LL-37 treatment to modulate the inflammatory response to influenza virus. They demonstrate that LL-37 treatment does not protect against influenza infection by promoting sustained inflammation, but suggest the possibility that cathelicidins may be protective by inhibiting excessive inflammation, or may have a direct anti-viral effect on influenza virus.

### Cathelicidins show antiviral activity against influenza virus *in vitro*

**Figure 4:**
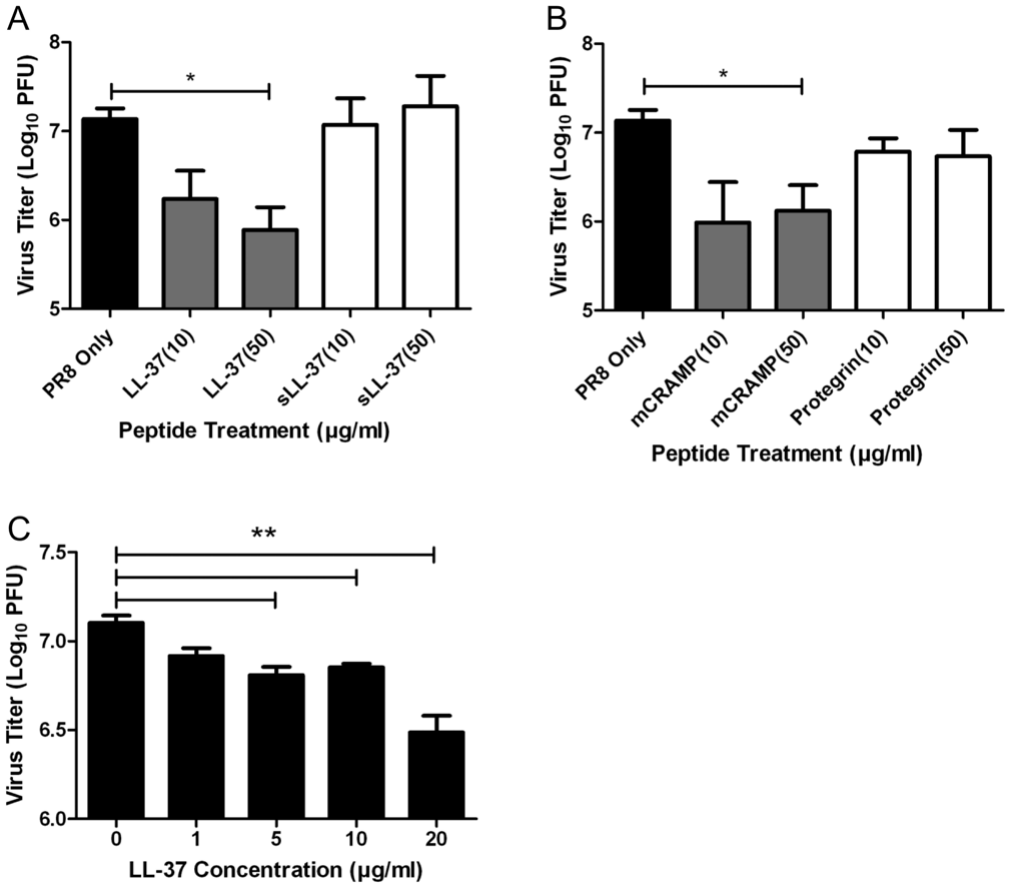
Cathelicidins show antiviral activity against influenza virus *in vitro.* Influenza virus was pre-incubated with cathelicidin peptide or control peptide scrambled LL-37 (sLL-37) (A) at a range of concentrations as indicated for 1 hour at room temperature and a plaque formation assay was then performed to assess virus titer in MDCK-L cells in the presence of trypsin. Viruses used were A/PR/8/34 (H1N1) (A, B) or A/Udorn/307/72 (C). The antiviral activity of the cathelicidins LL-37 (A, C), mCRAMP (B) and Protegrin-1 (B) was assessed. Figures are representative of at least three independent experiments. Figures show mean values ± SEM. Statistical analysis was performed using an unpaired t-test to compare virus only titer with virus + peptide (*P≤0.05, ** P≤0.01).

To assess the direct antiviral activity of cathelicidins *in vitro*, we incubated PR/8 with varying concentrations of cathelicidin peptides or control scrambled peptide, for 1 hour at room temperature and measured virus titer by plaque assay. Exposure to physiologically relevant concentrations of LL-37 led to approximately 90% reductions in virus titer (p≤0.05) (Figure 4A). Control scrambled LL-37 had no effect on the virus titer. The murine mCRAMP peptide also displayed anti-influenza virus activity and induced a significant decrease in virus titer following exposure (P≤0.05; Figure 4B). However, consistent with the *in vivo* observations, the porcine cathelicidin Protegrin-1 did not demonstrate any anti-influenza activity in this assay (Figure 4B). 

We also tested the effect of cathelicidins on a different subtype of influenza virus, A/Udorn/307/72 (H3N2). As with PR/8, exposure to physiologically relevant concentrations of LL-37 and mCRAMP caused a significant reduction in virus titer (P≤0.01; Figure 4C).

These data support the hypothesis that the antiviral effects of these cathelicidins are at least partially due to direct effects of the peptides upon the influenza virion.

### D-Isomer cathelicidins inhibit influenza virus

**Figure 5:**
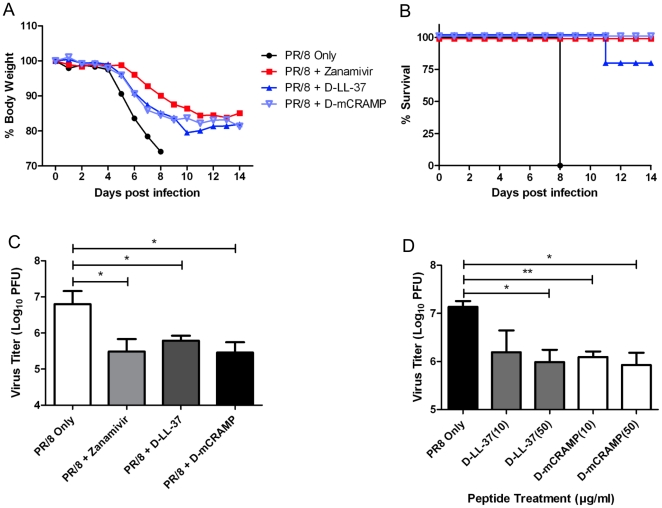
D-Isomer cathelicidins inhibit influenza virus. (A,B) Groups of 5 mice were infected with 10 MLD_50_ of A/PR/8/34 influenza virus via intranasal administration on day 0. Mice were nebulized with 200 µl of saline (control), zanamivir (500 µg/ml), D-LL-37 peptide (500 µg/ml) or D-mCRAMP peptide (500 µg/ml) once daily from day -1 to day 7. Mouse weight and survival was monitored daily up to 14 days post infection. Data represent mean values ± SEM from n = 1 experiment. Statistical analysis was performed using Kaplan Meier with a Mantel-Cox (log rank) test. Survival curves obtained with Zanamivir, D-LL-37 and D-mCRAMP treatments were significantly different (P≤0.001) compared to saline control treatment. (C) Groups of 5 mice were infected with 10MLD_50_ of A/PR/8/34 virus via intranasal administration on day 0. Mice were nebulized with 200 µl of saline (control), zanamivir (500 µg/ml), D-LL-37 peptide (500 µg/ml) or D-mCRAMP peptide (500 µg/ml) once daily from day -1 to day 2. Mice were euthanized on day 3 and viral titer in the lungs was assessed by plaque assay. Figure is representative of n = 3 independent experiments. Figure shows mean values ± SEM. Statistical analysis was performed using an unpaired t-test to compare virus infected animals with virus/peptide and virus/zanamivir treated animals (*P≤0.05). (D) The antiviral activity of the cathelicidins D-LL-37 and D-mCRAMP was assessed. Figure is representative of n = 3 independent experiments. Figure shows mean values ± SEM. Statistical analysis was performed using an unpaired t-test to compare PR/8 only titer with PR/8 + Peptide (*P≤0.05, ** P≤0.01).

To further test whether the anti-influenza activity observed with LL-37 and mCRAMP peptides was related to the physical properties of the peptide or whether interactions with a specific receptor might be involved, we synthesized LL-37 and mCRAMP peptides using only D-amino acids instead of L-amino acids and tested the antiviral properties of the peptides both *in vitro* and *in vivo*. Notably, both LL-37 and mCRAMP that had been synthesized with D-amino acids demonstrated effective antiviral activity *in vitro*, reducing titers as effectively as the L-peptides (Figure 5D). Consistent with this *in vitro* effect, the D-peptides were also effective *in vivo*. Mice were treated with nebulized D-LL-37 and D-mCRAMP for 1 day prior to and for 7 days post infection with 10MLD_50_ A/PR/8/34 (H1N1) virus. Mice were monitored for 14 days following infection. Mice that had received treatment with either of the D-analogs showed less body weight loss compared to control infected animals (Figure 5A) and survival was dramatically enhanced (Figure 5B). All control infected mice succumbed to PR/8 infection by day 8. However, there was significantly greater survival in the mice treated with D-LL-37 (80%) or D-mCRAMP (100%) (Figures 5A and 5B, P≤0.001). Additionally, lung virus titers assessed at 3dpi were markedly lower in mice treated with D-peptides compared with mice that had been treated with nebulized saline only (P≤0.05; Figure 5C). Weight loss, survival and lung titers in D-peptide treated mice were comparable to those in mice treated with zanamivir. These data suggest that the antiviral effects mediated by cathelicidins *in vivo* may be due to physical properties of the peptides rather than interactions with stereoisomer-specific receptors.

## Discussion

Many studies have demonstrated that cationic host defense peptides (CHDP, also known as antimicrobial peptides) can display potent activity against bacteria and viruses. In addition to the broad spectrum antibacterial potential, the cathelicidin family of peptides has previously been shown to display antiviral activity towards human immunodeficiency virus (HIV-1), vaccinia virus, adenovirus and herpes simplex virus-1 and -2 [6], [10], [26], [28]. In addition, the other major human family of CHDP, defensins, have also been reported to show anti-influenza virus properties [2], [3], [29].

In this study we demonstrate that human and murine cathelicidin peptides display antiviral activity against influenza virus *in vitro* and *in vivo* and that therapeutic administration of these peptides can provide significant protection against influenza virus infection in a mouse model. In addition to enhancing our understanding of the role of cathelicidins in innate host defense against influenza virus, these findings may point towards future therapeutic approaches for influenza virus infection.

Our data demonstrate that delivery of nebulized LL-37 peptide to mice infected with a lethal A/Puerto Rico/8/1934 (H1N1) influenza virus infection increased survival and decreased body weight loss when compared to either a scrambled control LL-37 peptide or saline control. Mice treated with the LL-37 peptide also displayed significantly reduced lung virus titers. Similarly, the mouse cathelicidin peptide, mCRAMP significantly reduced lung titers of PR8 and increased survival with reduced clinical disease (as measured by weight loss). The protective effects of LL-37 and mCRAMP were similar in magnitude to that of the well-characterized antiviral drug zanamivir.

The porcine neutrophil-derived cathelicidin peptide, Protegrin-1, did not appear to have any effect in the mouse model of infection. Previous studies in mice have demonstrated that Protegrin-1 shows potent antiviral activity towards HSV-1, HIV-1 and Lentivirus together with antibacterial activity towards *Haemophilus*, *Chlamydia* and *Actinobacillus* among others [30], [31], [32]. However, this activity did not extend to extend to influenza virus in either *in vitro* or *in vivo* experiments, indicating that that anti-influenza virus activity of cathelicidins is not inherent in all cathelicidin peptides. Further experiments will be needed to determine the reasons for this difference, and whether Protegrin-1 may be protective against swine-origin 2009 pandemic H1N1 human influenza viruses rather than the human-origin, mouse-adapted PR/8 virus.

LL-37 has been shown to have both direct microbicidal potential and a broad range of immunomodulatory properties; including direct [33], [34], [35] and indirect chemotactic functions [36], [37], and the capacity to modulate neutrophil function [38], the death of neutrophils [39], [40], [41] and infected epithelial cells [42], autophagy in infected macrophages [43], and the differentiation and function of dendritic cells [44], [45]. Many of these properties have the potential to influence the outcome of influenza infection *in vivo*, and dissecting the key mechanisms involved remains a future challenge. In particular, cathelicidins are capable of inducing chemokine and cytokine responses [36], [37], [46], and of modulating cytokine responses to pathogen-associated stimuli; including the inhibition of pro-inflammatory responses to lipopolysaccharide [36], [47], [48] and enhanced responses to IL-1β [49]. Infection with influenza virus induces expression of multiple pro-inflammatory cytokines, an effect that is essential for host defense against influenza virus infection [1]. In particular IL-1β has been shown to be a critical component of host defense against influenza in murine lung infection [50], [51], [52], induced in response to viral activation of the NLRP3 inflammasome, with influenza stimulating increased IL-1β production by macrophages [53]. Furthermore, influenza virus has been shown to employ multiple strategies to subvert the immune response [54], including suppression of IL-1β activity [55]. To test whether LL-37 modulated the inflammatory cytokine response to influenza, we measured cytokines in the bronchoalveolar lavage (BAL) fluid of mice following infection in the presence or absence of LL-37 treatment. As expected, PR/8 infection led to marked upregulation of multiple inflammatory cytokines three days after infection. However, this increase was not observed in LL-37-treated mice suggesting either that the inflammatory response was diminished by LL-37, or that virus replication was inhibited to the point that an inflammatory response was not induced or was resolved by day three after infection. Irrespective, these data suggest that LL-37 does not inhibit virus replication by inducing an enhanced inflammatory response over the course of the infection.

It seems likely that the decreased BAL fluid IL-1β concentration (and overall decrease in other inflammatory cytokines) observed in LL-37-treated infected mice is a consequence of peptide enhanced early clearance of the virus by day 3 as demonstrated in Figure 1C. However, the extent to which LL-37 might also directly modulate cytokines in response to infection remains to be determined. Nevertheless, these data demonstrate that therapeutic use of LL-37 provides enhanced protection against influenza virus, in the absence of prolonged upregulation of potentially harmful inflammatory cytokines in the lung [56],[57].

To examine whether cathelicidin peptides have antiviral activity against influenza virus in the absence of a cellular immune response, we tested their ability to reduce viral infectivity in cell culture. Exposure of two different influenza viruses (A/PR/8/34 H1N1 and A/Udorn/307/72 H3N2) to concentrations of LL-37 peptide naturally found in the inflamed human lung [58], [59] resulted in significant (p≤0.05) decreases in the number of plaque forming units in both viruses. As well as demonstrating that the antiviral effects of LL-37 were not limited to the PR/8 virus, these data demonstrate that LL-37 can have antiviral activity *in vitro* and indicate that LL-37 may have a direct effect on virus viability, or adversely influence viral adherence, internalization or replication, as shown for various defensins [2], [60]. The murine mCRAMP peptide also demonstrated significant anti-influenza activity, while the porcine cathelicidin Protegrin-1 and scrambled LL-37 peptide had no antiviral effect *in vitro*, correlating with the lack of protection observed *in vivo*. In contrast, both D-LL-37 and D-mCRAMP retained their potent antiviral activity *in vitro* and *in vivo,* although at the dosages used, there was no statistically significant advantage gained by using the metabolically more stable D-analogs. This further supports the suggestion that physical properties of the cathelicidin peptides, rather than interactions with receptors, are involved in the antiviral effects of these peptides.

In summary, this study demonstrates that cathelicidins possess potent antiviral activity against influenza virus. This antiviral activity is at least partially mediated by a direct effect on the virion, with an as yet undetermined contribution from cathelicidin-mediated immunomodulation. These data suggest that approaches aimed at increasing natural cathelicidin expression in the influenza-infected lung, or therapeutic treatment of influenza-infected individuals may provide significant protection against disease. Such approaches may include increasing vitamin-D levels to boost endogenous cathelicidin production or the therapeutic administration of naturally-occurring cathelicidins or synthetic analogues such as D-form peptides (which show greater stability against proteases) or peptides modified to maximize the key anti-influenza functions.

## Materials and Methods

### Animals & Ethics Statement

6 to 8 week old female BALB/c mice were supplied by Jackson Laboratories, USA and housed and handled per approved protocols in compliance to the CDC Institutional Animal Care and Use Committee guidelines (IACUC 2064DONMOU).

### Viruses and Cells

A/Puerto Rico/8/1934 (H1N1) and A/Udorn/307/72 (H3N2) viruses were propagated and titrated as described previously [61]. Madin Darby Canine Kidney (MDCK) cells were obtained from the Amerian Type Culture Collection (Manassas, VA).

### Infection and Nebulization

Mice were lightly anaesthetized with 1.5% isoflurane and inoculated with 10 median lethal doses (MLD_50_) of A/Puerto Rico/8/1934 (H1N1) (approximately 350 plaque forming units) by intranasal instillation of 50 µl of virus suspension. For nebulization, mice were briefly anaesthetized with 1.5% isoflurane and placed into a cylindrical exposure chamber with their nose protruding through a nasal cone at the top of the chamber. A nebulizer unit (Kent Scientific, CN, USA) was connected to the top of the exposure chamber and mice were exposed to either CHDP suspended in endotoxin-free water as indicated, or water-only control for 30 seconds. Mice were then removed from exposure chamber and allowed to recover. Mice were monitored daily until 14 d.p.i for mortality and weight loss. Mice that lost >25% of their initial body weight were euthanized in accordance with our approved animal study protocol.

### Viral Titer Assessment

Mouse lung viral titers were measured 3 days post infection. Briefly, mice were euthanized 3 days post infection and tissues were extracted and stored at −80°C. Lung tissue was homogenized in 1 ml ice cold PBS using a MagNA Lyser tissue homogenizer (Roche, IN, USA). Homogenates were clarified by centrifugation at 10,000 x g for 5 minutes and the viral titer of the supernatant was assessed by plaque formation assay in MDCK-L cells in the presence of trypsin [61].

### Cytokine Analysis

Measurement of cytokine concentration in the bronchoalveolar lavage fluid was performed via Bioplex analysis. Briefly, mice were euthanized 3 days post infection and bronchoalveolar lavage was performed *in situ* using 1 ml sterile ice cold PBS. Samples were aliquoted and frozen at −80°C until use. Cytokine analysis was then performed using a BioPlex Mouse Cytokine Assay Kit (Bio-Rad, CA, USA) according to manufacturer's instructions.

### Peptide Synthesis and Purification

The peptides were assembled using the Fmoc/tBu solid-phase peptide synthesis approach [62] using either model 433A (Applied Biosystems, CA, USA) or model Liberty (CEM Corporation, NC, USA) automated peptide synthesizers followed by cleavage in the trifluoroacetic acid (TFA) / phenol / thioanisole / ethanedithiol/water (10∶0.75∶0.5∶0.25∶0.5, w/w) mixture at 25°C for 90 min followed by precipitation with cold diethyl ether. The crude peptides were purified by preparative reversed-phase high-pressure liquid chromatography (RP-HPLC). The peptide purity (>98%) was confirmed by analytical RP-HPLC, and the masses were confirmed by mass spectrometry. Following lyophilization, the purified peptides were obtained in the form of their TFA salts; namely: LL-37 (LLGDFFRKSKEKIGKEFKRIVQRIKDFLRNLVPRTES), LL-37 analog having “scrambled” sequence (RSLEGTDRFPFVRLKNSRKLEFKDIKGIKREQFVKIL), termed sLL-37 (control peptide), mouse cathelicidin mCRAMP (GLLRKGGEKIGEKLKKIGQKIKNFFQKLVPQPEQ), and their all-D-amino acid residues containing analogs, D-LL-37 and D-mCRAMP. Since porcine Protegrin-1 (PG-1; RGGRLCYCRRRFCVCVGR –amide) was obtained in its reduced form, its two disulfide bridges, connecting Cys-6 and Cys-15, and Cys-8 and Cys-13, were formed by air oxidation of the HPLC purified, all-reduced peptide. The antimicrobial activity of the preparations of LL-37, mCRAMP, and Protegrin-1 peptides was confirmed in the antibacterial assays against *Neisseria gonorrhoeae* (FA19), *Acinetobacter baumannii,* and *Staphylococcus aureus* (COL) (data not shown).

### In Vitro Antiviral Activity of Cathelicidins

To assess *in vitro* effects of peptides on influenza virus, A/Puerto Rico/8/1934 (H1N1) virus and A/Udorn/307/72 (H3N2) were exposed to peptides for 1 hour at room temperature in DMEM. Ten-fold serial dilutions in DMEM were then performed to assess the resulting viral titer by plaque formation assay in MDCK-L cells in the presence of trypsin in the agarose overlay.

### Statistical Analysis

Statistical analysis of the data was performed using GraphPad Prism software. Survival curves were analysed using Kaplan Meier with a Mantel-Cox (log rank) test. Weight loss between treatment groups was assessed at day 7 by an unpaired t-test comparing virus infected animals with virus/peptide and virus/zanamivir treated animals. Statistical analysis of the alterations in lung virus titer as a result of peptide treatment was performed using an unpaired t-test to compare virus infected animals with virus/peptide and virus/zanamivir treated animals.
